# Supplementary material for: Cross-talk between QseBC and PmrAB two-component systems is crucial for regulation of motility and colistin resistance in Enteropathogenic Escherichia coli
Source: PLoS Pathog. 2023 Dec 7;19(12):e1011345. doi: 10.1371/journal.ppat.1011345 (PMC10729948; doi:10.1371/journal.ppat.1011345)
Supplement: S1 Table — Growth rate was calculated by fitting growth curves into a logistic growth equation (n = 3). (PDF) [file ppat.1011345.s001.pdf]

Strain	Growth rate ± SD	Strain	Growt rate ± SD
WT	0.7891 ± 0.0370	Δ*pmrB*Δ*qseB*	0.8090 ± 0.0292
Δ*qseB*	0.7978 ± 0.0390	Δ*pmrB*Δ*qseC*	0.6879 ± 0.0300
Δ*qseC*	0.5798 ± 0.0550	Δ*kdpD*	0.8859 ± 0.0372
Δ*qseB*Δ*qseC*	0.8493 ± 0.0399	Δ*kdpE*	0.7075 ± 0.0356
Δ*pmrA*	0.6975 ± 0.0368	Δ*kdpD* Δ*qseB*	0.7896 ± 0.0335
Δ*pmrB*	0.6975 ±0 .0335	Δ*kdpD* Δ*qseC*	0.5925 ± 0.0547
Δ*pmrA*Δ*pmrB*	0.7455 ± 0.0390	Δ*kdpE* Δ*qseB*	0.8755 ± 0.0387
Δ*pmrA*Δ*qseB*	0.8208 ± 0.0335	Δ*kdpE* Δ*qseC*	0.5099 ± 0.0290
Δ*pmrA*Δ*qseC*	0.4132 ± 0.0368		
