# Supplementary material for: Cross-talk between QseBC and PmrAB two-component systems is crucial for regulation of motility and colistin resistance in Enteropathogenic Escherichia coli
Source: PLoS Pathog. 2023 Dec 7;19(12):e1011345. doi: 10.1371/journal.ppat.1011345 (PMC10729948; doi:10.1371/journal.ppat.1011345)
Supplement: S2 Table — Individual values of four replicates are represented in the peak-luminescence of each strain and each gene. (PDF) [file ppat.1011345.s002.pdf]

**RIL X100**	**Strain**																	
	WT	Δ*qseB*	Δ*qseC*	WT	Δ*qseB* Δ*qseC*	Δ*pmrA*	WT	Δ*pmrB*	Δ*pmrA* Δ*pmrB*	WT	Δ*pmrA* Δ*qseb*	Δ*pmrA* Δ*qseC*	WT	Δ*pmrB* Δ*qseB*	Δ*pmrB* Δ*qseC*	WT	Δ*kdpD*	Δ*kdpE*
flhCD	346.848	553.305	248.169	162.472	269.325	158.306	296.049	321.288	239.009	214.025	349.089	195.154	269.004	399.367	297.976	247.218	203.503	209.732
	441.918	505.82	235.308	266.621	386.239	229.128	333.314	302.632	284.732	305.453	378.18	155.708	333.151	446.96	262.798	276.303	284.488	259.57
	252.738	420.54	207.606	338.353	413.034	275.358	339.441	229.744	356.407	297.958	364.17	202.618	240.01	310.457	250.777	246.372	229.833	229.039
	310.744	480.617	203.454	333.78	509.053	188.581	211.788	201.032	342.699	351.755	402.452	111.647	290.156	343.573	250.829	272.253	263.416	235.525
fliA	959.862	3072.369	138.495	542.86	1449.421	664.318	989.394	803.131	958.195	764.477	2010.685	131.404	834.926	2498.183	1107.723	631.048	695.399	745.913
	520.183	1971.85	96.818	353.587	866.32	270.725	535.144	531.27	460.432	391.612	1585.187	56.107	557.995	2609.896	516.662	379.924	253.463	373.258
	796.053	2647.081	99.691	885.882	2951.784	657.2	607.745	653.092	989.744	686.32	2357.655	45.52	659.676	1761.006	462.603	688.05	821.654	774.962
	469.19	1350.341	70.828	442.941	2173.476	356.703	296.393	256.1	505.564	432.051	1630.952	49.659	379.625	1517.102	302.594	428.292	267.253	368.893
bla	299.871	277.039	244.887	120.816	147.876	149.492	213.456	170.201	227.147	191.384	183.154	60.376	217.95	202.876	173.705	180.778	190.622	159.61
	210.943	210.55	240.537	261.585	257.105	191.562	230.035	166.194	147.255	170.321	166.242	85.386	199.269	209.391	161.973	176.868	209.594	136.751
	207.606	216.204	216.503	124.95	133.115	151.155	190.496	175.608	250.67	243.497	229.496	90.835	180.438	180.313	143.327	209.874	187.088	174.844
	201.378	204.699	210.096	242.74	249.543	197.596	205.879	222.584	288.611	208.82	212.544	76.559	176.29	178.24	134.873	183.999	208.734	139.106
ler (LB)	827.97	1145.716	873.108	501.261	659.48	409.394	722.526	732.193	591.147	760.213	945.204	622.831	774.239	1101.524	684.061	850.825	821.773	772.849
	606.88	1156.02	432.416	505.421	510.816	342.893	630.139	666.25	437.656	586.002	766.157	373.072	608.627	1006.458	556.873	614.254	623.667	580.623
	750.564	1111.197	737.112	948.897	1373.117	655.29	704.902	604.619	925.594	850.573	1038.442	843.289	770.998	1159.365	609.755	831.845	844.214	906.061
	618.234	940.456	502.482	788.97	1267.226	657.06	667.774	549.966	708.683	708.136	910.426	514.654	624.273	825.633	495.343	768.451	603.746	536.182
ler (DMEM)	1800.338	1604.525	2383.4	1840.498	1941.178	1631.242	1341.691	1303.117	1425.165	1752.953	1752.979	1677.735	1277.112	1628.928	1459.776	857.425	906.656	795.03
	1613.97	1441.61	2351.521	1461.687	1555.718	1563.852	1132.725	1015.939	1421.499	1301.516	1343.736	1400.829	1050.55	1380.439	1333.611	452.442	485.263	299.617
	1272.468	1704.648	1127.916	1571.987	1443.869	1097.377	1752.979	1645.047	1850.678	1466.239	1372.692	998.506	1704.172	1262.877	1125.466	514.779	590.568	609.859
	937.498	1321.005	1081.885	1125.557	1355.97	947.666	1404.805	1244.574	1643.163	1319.267	1068.464	913.496	1431.415	1121.479	1095.341	403.153	433.172	460.916
recA	941.713	1498.086	545.91	511.773	805.928	485.223	821.808	866.953	920.945	741.173	1026.2	264.606	807.017	1433.998	730.652	672.359	707.638	702.555
	1248.748	1697.968	444.586	603.368	1029.937	530.38	900.669	974.546	883.506	849.527	1190.169	301.043	972.559	1628.519	846.892	806.651	808.308	746.076
	907.099	1333.848	404.69	1034.871	1626.662	573.56	906.107	784.109	1166.92	791.749	1197.408	229.671	750.288	1157.469	636.857	683.262	678.217	711.243
	997.809	1311.171	373.56	1045.54	1765.654	756.349	738	706.079	1061.531	879.476	1359.143	335.196	735.82	1457.171	663.22	720.662	847.245	633.933
