# Supplementary material for: Cross-talk between QseBC and PmrAB two-component systems is crucial for regulation of motility and colistin resistance in Enteropathogenic Escherichia coli
Source: PLoS Pathog. 2023 Dec 7;19(12):e1011345. doi: 10.1371/journal.ppat.1011345 (PMC10729948; doi:10.1371/journal.ppat.1011345)
Supplement: S3 Table — (PDF) [file ppat.1011345.s003.pdf]

Plasmid name	Relevant features[a]	Reference
pCas	*repA101*(Ts) *kan* P_{cas} – *cas9* P_{araB} – *Red* *lacI*^{q} P_{trc} – sgRNA-*pMB1*	[33]
pTargetF-*cadA*	*pMB1 aadA* P_{J23119} - sgRNA-*cadA*	[33]
Derivatives from pTargetF-*cadA*		
pTargetF-*qseB*	*pMB1 aadA* P_{J23119} – sgRNA-*qseB*	This study
pTargetF-*qseC*	*pMB1 aadA* P_{J23119} – sgRNA-*qseC*	This sutdy
pTargetF-*pmrA*	*pMB1 aadA* P_{J23119} – sgRNA-*pmrA*	This study
pTargetF-*pmrB*	*pMB1 aadA* P_{J23119} – sgRNA-*pmrB*	This study
pTargetF-*kdpD*	*pMB1 aadA* P_{J23119} – sgRNA-*kdpD*	This study
pTargetF-*kdpE*	*pMB1 aadA* P_{J23119} – sgRNA-*kdpE*	This study
pXen-1	*pMB1 bla cam* promotorless *luxABCDE*	[51]
pTarget_lux	*pMB1 aadA* P_{J23119} - *luxAB*	This study
Derivatives of pTarget_lux		
pTarget_lux-*flhC*	*pMB1 aadA* P_{flhC} – *luxAB*	This study
pTarget_lux-*fliA*	*pMB1 aadA* P_{flliA} – *luxAB*	This study
pTarget_lux-*bla*	*pMB1 aadA* P_{bla} – *luxAB*	This study
pTarget_lux-*ler*	*pMB1 aadA* P_{ler} – *luxAB*	This study
pTarget_lux-*recA*	*pMB1 aadA* P_{recA} – *luxAB*	This study

[a]Ts, temperature sensitive; *kan* kanamycin resistance gene; P_{cas} – *cas9, cas9* gene with its native promoter; P_{araB} – *Red,* the *λ-red* recombination system with an arabinose-inducible promoter; *lacI*^{q}*, lacI* repressor, P_{trc} – sgRNA-*pMB1* sgRNA with a 20-bp CRIPSR guide targeting *pMB1* origin of replication, under the control of *trc* promoter; P_{J23119} - sgRNA-*cadA,* sgRNA with a 20-bp promoter targeting *cadA* gene, under the control of the constitutive promoter J23119; *bla* ampicillin resistance gene; *aadA*, spectinomycin resistance gene.
