# Supplementary material for: Cross-talk between QseBC and PmrAB two-component systems is crucial for regulation of motility and colistin resistance in Enteropathogenic Escherichia coli
Source: PLoS Pathog. 2023 Dec 7;19(12):e1011345. doi: 10.1371/journal.ppat.1011345 (PMC10729948; doi:10.1371/journal.ppat.1011345)
Supplement: S4 Table — (PDF) [file ppat.1011345.s004.pdf]

Primer	Sequence 5'-3'	Used for:
pBM001	TTACTGTCGGTGCTGAAGTTAG	HdsR_Fw
pBM002	TGGTCGTGAATTGCATTCG	HdsR_Rv
pBM003	CTGTGCGGTATTTCACACC	Guide sequencing
pBM004	TGAACTCGAGTAGGGATAACAGG	Amplify pTarget Rv for guide introd.
pBM005	CCGACTAGTCGTTGATAGAAGTCGCCGCCGTTTTAGAGCTAGAAATAGCAAG	QseB Guide Fw
pBM007	TTGCGGATCTGTTTGACGTC	QseB_LHA_Fw
pBM008	CCTAATGTGTAGCCAATACCATGAGTAAAATTCGCATTTTTTCATCC	QseB_LHA_Rv
pBM009	GGATGAAAAAATGCGAATTTTACTCATGGTATTGGCTACACATTAGG	QseB_RHA_Fw
pBM010	GTCTTCGCGGTATTCCCATTC	QseB_RHA_Rv
pBM011	TGCGAAATTATTCCCGGGAAC	Check QseB mutation Fw
pBM012	ACAAGCCACGGATCAATTG	Check QseB mutation Rv
pBM013	CCGACTAGTGGTGATGGATATTTACCACAGTTTTAGAGCTAGAAATAGCAAG	QseC Guide Fw
pBM015	GGTTAAGTACGCTCGATCTCAAC	QseC_LHA_Fw
pBM016	CCAGCTTACCTTCGTCTCAATTCAACGACTCAACCAGTGG	QseC_LHA_Rv
pBM017	CCACTGGTTGAGTCGTTGAATTGAGACGAAGGTAAGCTGG	QseC_RHA_Fw
pBM018	ACTGATCTTTTTCGGTGAAGGC	QseC_RHA_Rv
pBM019	TTTCCAGCTTTGTCGCCTG	Check QseC mutation Fw
pBM020	GGCAGATACACACCGTCAAC	Check QseC mutation Rv
pBM021	CCGACTAGTCGGGCGGTGAGAATTAACACGTTTTAGAGCTAGAAATAGCAAG	PmrA Guide Fw
pBM023	GACGTCAGCGGTAAACAACC	PmrA_LHA_Fw
pBM024	GCAGGGATTGATTCTGGCGATTTGGCTATATGCTGGTCGC	PmrA_LHA_Rv
pBM025	GCGACCAGCATATAGCCAAATCGCCAGAATCAATCCCTGC	PmrA_RHA_Fw
pBM026	CAGGAAATTTACCCCAACCTGC	PmrA_RHA_Rv
pBM027	TTCCAGATGCAAACGCACC	Check PmrA mutation Fw
pBM028	CCTATTACAACCGCTATCCGC	Check PmrA mutation Rv
pBM029	CCGACTAGTTCGCAATAACATCCGCAGCAGTTTTAGAGCTAGAAATAGCAAG	PmrB Guide Fw
pBM031	ATATTTCGGTGCTGGTTGCC	PmrB_LHA_Fw
pBM032	CTGGTTTGCCACGTACTGATCGACGTCAGCGGTAAACAACC	PmrB_LHA_Rv
pBM033	GGTTGTTTACCGCTGACGTCGATCAGTACGTGGCAAACCAG	PmrB_RHA_Fw
pBM034	ATTTGGCTATATGCTGGTCGC	PmrB_RHA_Rv
pBM035	ACGCATATCCGTTACAGCG	Check PmrB mutation Fw
pBM036	GCATATCCATAACCTGCGCG	Check PmrB mutation Rv

Primer	Sequence 5'-3'	Used for:
pBM037	TCCGACTAGTGTAGATTTTCGCCCTCAACGGTTTTAGAGCTAGAAAT AGCAAG	KdpD Guide Fw
pBM039	TCACACGAGCTGCTAAATCG	KdpD_LHA_Fw
pBM040	CGTAGTTCGCAGGATATCGCCCAAACAGCCCCTGAACTTGAAG	KdpD_LHA_Rv
pBM041	CTTCAAGTTCAGGGGCTGTTTGGGCGATATCCTGCGAACTACG	KdpD_RHA_Fw
pBM042	CGATCTCGATCAGGTGCTGG	KdpD_RHA_Rv
pBM043	CAGCAGGCGGAACTCAATC	Check KdpD mutation Fw
pBM044	TGGCGTCGGGAAACATTTG	Check KdpD mutation Rv
pBM045	TCCGACTAGTACATCGGAAAATTTCACCAGGTTTTAGAGCTAGAAAT AGCAAG	KdpE Guide Fw
pBM047	AACAGAGAATGAGCACTGCG	KdpE_LHA_Fw
pBM048	GAACAGGCTATTCGTCGCTTTGAAACCGGTATTGGGTATCG	KdpE_LHA_Rv
pBM049	CGATACCCAATACCGGTTTCAAAGCGACGAATAGCCTGTTC	KdpE_RHA_Fw
pBM050	ATGCTGGAACCGGGTTTATC	KdpE_RHA_Rv
pBM051	ACCCTTATCAGTCTGGTGGC	Check KdpE mutation Fw
pBM052	TAACGCTGGAAGAAGTGGTC	Check KdpE mutation Rv
pBM053	GCGTCACACTTTGCTATGC	Check pCas Fw
pBM054	GCAATGGTTTCAACCATGTACC	Check pCas Rv
pBM055	AATTACTAGTTTGCAGATGAAGCAAGAGG	Amplify luxAB SpeI Fw
pBM056	AATTGAATTCCATCCTCCTCCTCGACTTAGG	Amplify luxAB EcoRI Rv
pBM057	CTGTGCGGTATTTCACACC	Check promoter insert. Fw
pBM058	TGATTCAGCCACCACATAAAC	Check promoter insert. Rv
pBM059	AATTACTAGTTCAGCAACTCGGAGGTATGC	FlhCD Promoter Fw
pBM060	AATTGGATCCAGCATCGGCGCAGCTAATTG	FlhCD Promoter Rv
pBM061	AATTACTAGTCACCTTCAGCGGTATAGAGTG	FliA Promoter Fw
pBM062	AATTGGATCCCTGATTAACTGAGACTGACGG	FliA Promoter Rv
pBM063	AATTACTAGTGGGCGACACGGAAATGTTG	Bla Promoter Fw
pBM064	AATTGGATCCATGAATGATCGACCAGGCAATG	Bla Promoter Rv
pBM065	AATTACTAGTGCTGAATGTATGGACTTGTTG	LEE Promoter Fw
pBM066	AATTGGATCCGCCGAATGGATATGGGCAATAC	LEE Promoter Rv
pBM067	AATTACTAGTTCTCAATCTGGCCCAGTGC	RecA Promoter Fw
pBM068	AATTGGATCCAGCCTGTCGTGGTGGAAATG	RecA Promoter Rv
pBM069	TCCGACTAGTAAAAATGCGAATTTTACTCAGTTTTAGAGCTAGAAAT AGCAAG	QseB Complementation Guide
pBM070	TCCGACTAGTCAATTCAACGACTCAACCAGGTTTTAGAGCTAGAAAT AGCAAG	QseC Complementation Guide
pBM071	TCCGACTAGT CGCTGACGTCGATCAGTACG GTTTTAGAGCTAGAAATAGCAAG	PmrB Complementation Guide

Primer	Sequence 5'-3'	Used for:
pBM072	ATTCTGGCGTTCACATTGCCAGGCATGGATGGTCGCG	QseB Alanine change Fw
pBM073	GGCAATGTGAACGCCAGAATCACCGCATCATAAGGCGC	QseB Alanine change Rv
pBM074	TCCGACTAGTATCCTAGATTTAACCTTACCGTTTTAGAGCTAGAAATAGCAAG	QseB Alanine change guide
pBM075	CAGATGTGGCAGCTGAGCTTCGAACGCCACTGGCGG	PmrB Alanine change Fw
pBM076	AAGCTCAGCTGCCACATCTGCGGTAAACAACCTTTCGTTATCC	PmrB Alanine change Rv
pBM077	TCCGACTAGTCAGTTCGTGCGCGACGTCAGGTTTTAGAGCTAGAAATAGCAAG	PmrB Alanine change guide
