# Supplementary material for: Cross-talk between QseBC and PmrAB two-component systems is crucial for regulation of motility and colistin resistance in Enteropathogenic Escherichia coli
Source: PLoS Pathog. 2023 Dec 7;19(12):e1011345. doi: 10.1371/journal.ppat.1011345 (PMC10729948; doi:10.1371/journal.ppat.1011345)
Supplement: S5 Table — (PDF) [file ppat.1011345.s005.pdf]

**Primer**	**Sequence 5’-3’**
qPCR_GyrA_Ecoli_Fw	GTAACCCTGGTGAGAAAGCG
qPCR_GyrA_Ecoli_Rv	TCGTCTGATGGAAGTGATCCG
qPCR_ArnB_Fw	TGCTGAAATTTCACGGTCTGG
qPCR_ArnB_Rv	GACTAACTGTGTCAGGGCAATC
qPCR_EptA_Fw	TCCACGGGCTGGAAGATTAC
qPCR_EptA_Rv	GGTCTGGATCTCGTTAGTGTCG
qPCR_FlhCD_Fw	CGGTGATCAAAGCCTACCG
qPCR_FlhCD_Rv	GGTAATAAAATTGCCGCCGC
qPCR_FliA_Fw	AATGGATAAACACTCGCTGTGG
qPCR_FliA_Rv	AGCGTTCGACGGCATTAAG
qPCR_QseB_Fw	ATTACTGATGCGTAACGCTGG
qPCR_QseB_Rv	CCAATACCATGCACGGTACG
qPCR_lee_Fw	GCGAGAGCAGGAAGTTCAAAG
qPCR_lee_Rv	GCCCTTCTTCATTGCGGTAG
qPCR_EspA_Fw	GGGAGCATGTCGAAAGATGAC
qPCR_EspA_Rv	CCCAACCATTGCACCGATATG
