# Supplementary figures and images for: Cross-talk between QseBC and PmrAB two-component systems is crucial for regulation of motility and colistin resistance in Enteropathogenic Escherichia coli
Source: PLoS Pathog. 2023 Dec 7;19(12):e1011345. doi: 10.1371/journal.ppat.1011345 (PMC10729948; doi:10.1371/journal.ppat.1011345)

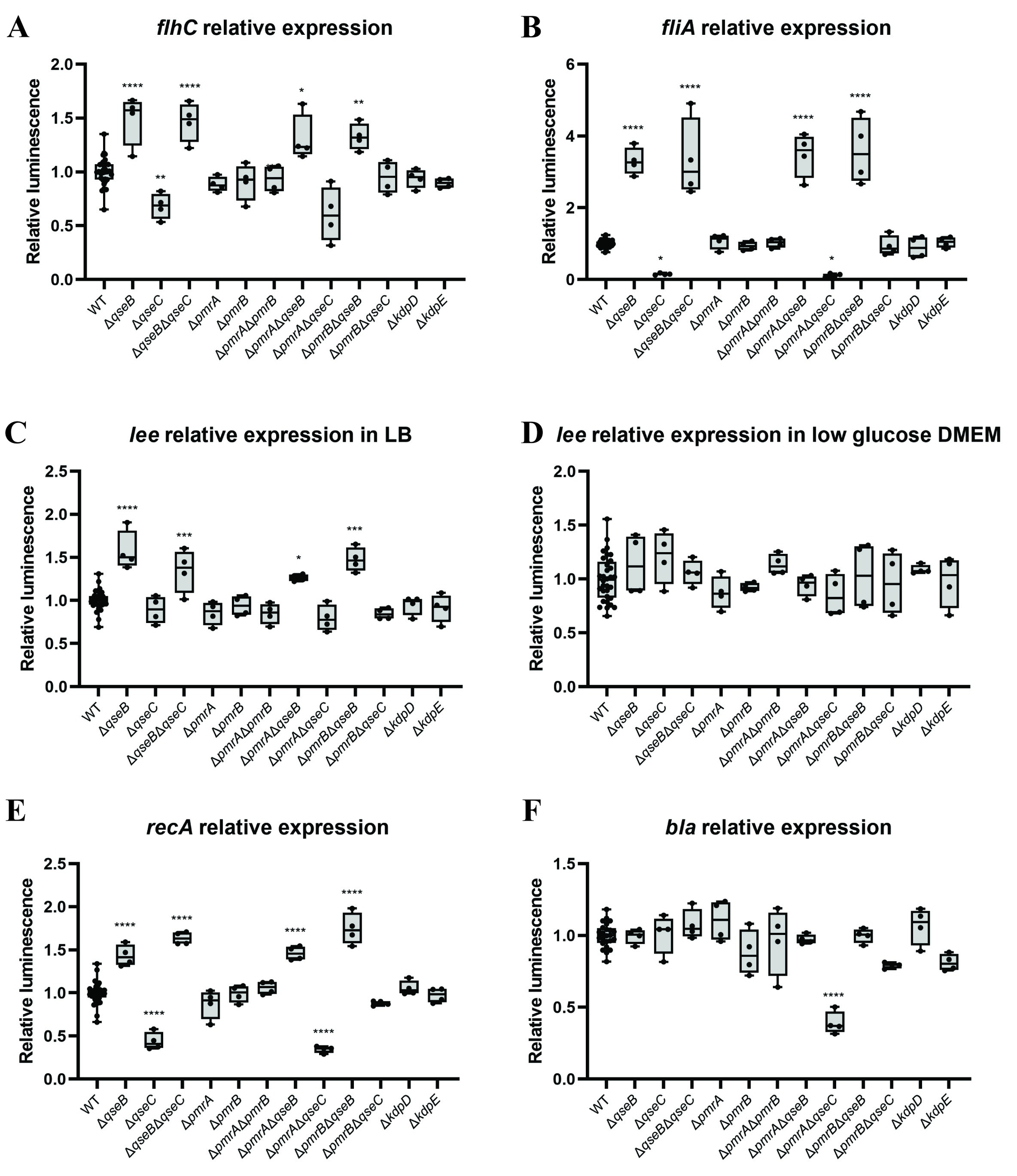

Supplement: S1 Fig — Differential expression according to the luciferase assay of the genes flhC, fliA, ler in LB or low glucose DMEM and recA of all tested mutants is depicted (A, B, C, D, E and F). * p-value <0.05; ** <0.01; *** <0.001; **** <0.0001 via one-way ANOVA with multiple comparisons, n = 4. (TIF) [file ppat.1011345.s006.tif]

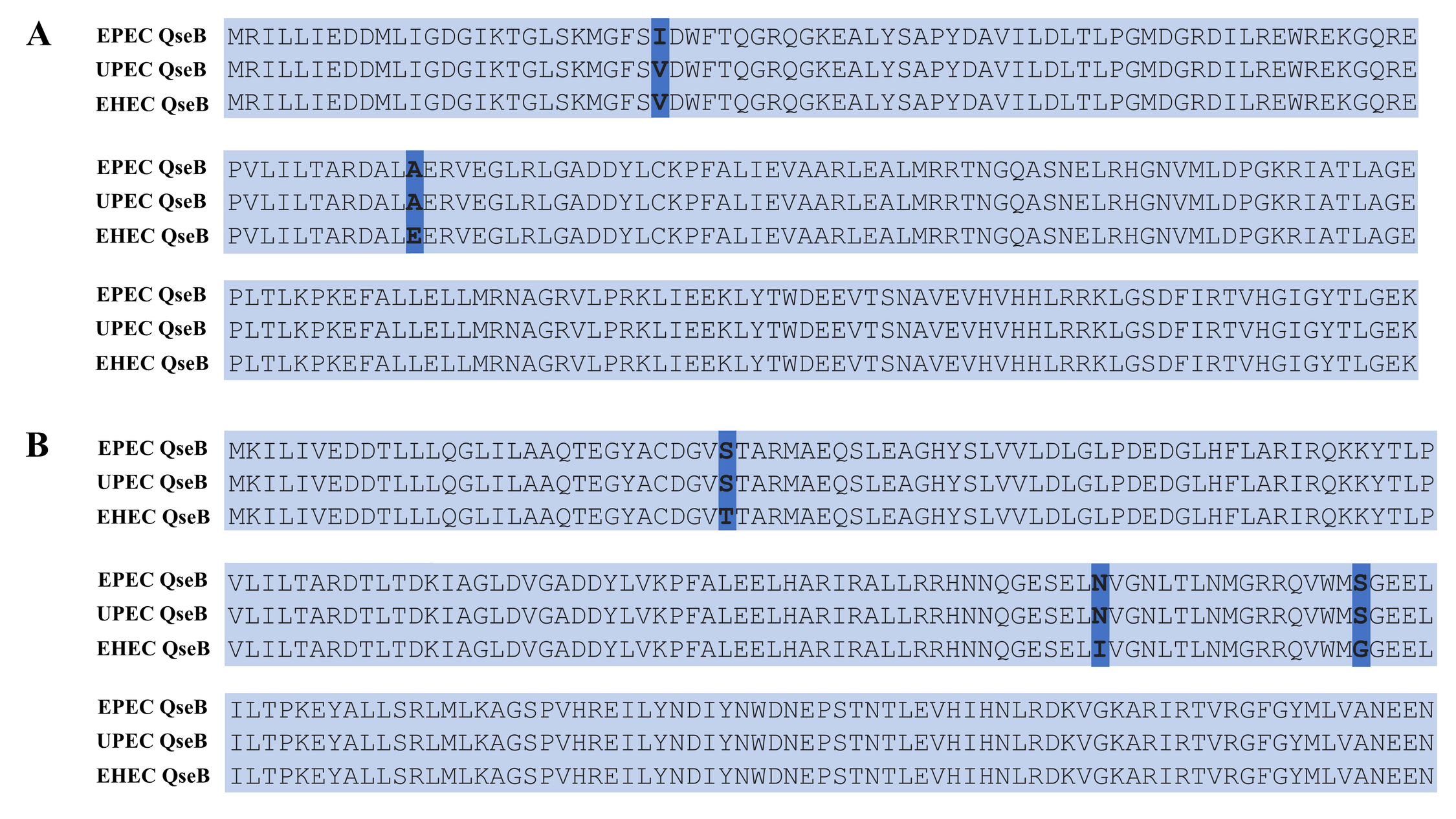

Supplement: S2 Fig — Light blue shows identical amino-acids, dark blue shows changes in the amino-acid sequence. (TIF) [file ppat.1011345.s007.tif]
