# Supplementary material for: Cross-talk between QseBC and PmrAB two-component systems is crucial for regulation of motility and colistin resistance in Enteropathogenic Escherichia coli
Source: PLoS Pathog. 2023 Dec 7;19(12):e1011345. doi: 10.1371/journal.ppat.1011345 (PMC10729948; doi:10.1371/journal.ppat.1011345)
Supplement: Attachment — Submitted filename: Rebuttal.pdf [file ppat.1011345.s008.pdf]

Dear Editor

Thank you for your interest and comments of the reviewers which helped us to improve the manuscript. We performed additional experiments to address some of the points raised by the reviewers and academic editor and provide a point-by-point rebuttal for your consideration.

Reviewer 1.

Comment 1. Does the *in vitro* and *in vivo* QseB phosphorylation in the absence of QseC is PmrB-dependent?

Using site-directed mutagenesis we have introduced a substitution of the conserved histidine residue in PmrB to an alanine, which is known to the prevent phosphotransfer to the response regulator. Introduction of this single amino-acid change in PmrB in a Δ*qseC* background *in vivo* recovers WT motility phenotypes, suggesting that indeed phosphorylation of QseB is dependent on PmrB in the absence of *qseC.* Besides, introduction of a non-phosphorylatable QseB (QseBD51A) in a Δ*qseC* background also recovers WT phenotypes, indicating that phosphorylation of QseB is the responsible of phenotypes seen in the Δ*qseC* background (Lines 160 to 174, Figure 4). In addition, Guckes *et al.*[1] showed in Uropathogenic *Escherichia coli* the cross-regulation between QseBC and PmrAB and that *in vitro* PmrB phosphorylates QseB. This is similar to the regulation we describe in EPEC. Given the new genetic data we provided we think the proposed *in vitro* phosphotransfer assay with PmrB and QseB is not critical. Besides this assay would require not only protein purification but also radioactive labelling of the phosphate which is not possible without a training course and purchase of new equipment.

[1] Guckes KR, Kostakioti M, Breland EJ, Gu AP, Shaffer CL, Martinez CR, et al. Strong cross-system interactions drive the activation of the QseB response regulator in the absence of its cognate sensor. Proceedings of the National Academy of Sciences. 2013 Oct 8;110(41):16592–7.

Comment 2. Should be analysed which motility genes (*fliC, flhCD*) are affected by transcriptional talk. Is there a consensus site for QseB-P in the motility genes? Does QseB-P bind to these motility regions?

We searched for QseB and PmrA motifs in EPEC using MEME suite, FIMO tool[1,2]. Consensus sites for QseB-P and PmrA were identified in the *flhCD* promoter, indicating that regulation is likely by direct interaction with the master regulon of the flagella *flhCD.* Later motility genes such as *fliA* are probably upregulated as consequence of upregulation of *flhCD* (Lines 323 to 328). This is supported by previous research from Clarke and Sperandio[3] that shows QseB binding to the *flhCD* promoter in the same region as the one predicted by MEME (-650 to -900 bp). Given the evidence provided in literature for this binding, we chose to show QseB-P binding to the same motif in the *arn* promoter which is addressed in comment 3 below.

[1] Bailey TL, Johnson J, Grant CE, Noble WS. The MEME Suite. Nucleic Acids Research. 2015 43(W1):W39-W49

[2] Grant CE, Bailey TL, Noble WS. FIMO: Scanning for occurrences of a given motif, Bioinformatics. 2011 27(7):1017-1018

[3] Clarke MB, Sperandio V. Transcriptional regulation of *flhDC* by QseBC and σ^{28} (FliA) in Enterohaemorrhagic *Escherichia coli*. Mol Microbiol. 2005 Sep;57(6):1734–49.

Comment 3. Should be corroborated if the QseB binding site found in arn (and eptA) is recognized by QseB-P (site-directed mutagenesis). This also applies for motility genes.

MEME suite was also able to identify consensus binding sites for QseB-P and PmrA in the *arn* promoter. We synthetized QseB and PmrA regulators (Material and methods, lines 510 to 513), however precipitation of purified PmrA protein rendered it unsuitable for further experiments. Thus electrophoretic mobility shift assays (EMSA) could only be performed with purified phosphorylated and non-phosphorylated QseB. We found that phosphorylated QseB indeed binds to the predicted QseB binding site in the *arn* promoter whereas the non-phosphorylated QseB binds with lower affinity. These assays corroborate that QseB directly regulates *arn* operon (Lines 265 to 269, Figure 8). A QseB binding site for *eptA* gene was not found. Upregulation of *eptA* in the absence of *qseC* and *pmrA* is probably indirect.

Reviewer 2.

Comment 1. Authors do not provide complementation in their studies. They should consider complementing with WT QseB or a phosphomimetic to validate their results.

In order to prove that off-target effects are not responsible for the observed phenotypic changes we complemented Δ*qseB* with *qseB* WT gene. This strain recovers WT phenotypes as predicted. Then, we complemented Δ*pmrB*Δ*qseC* first with WT *pmrB*, showing phenotypes similar to Δ*qseC* mutant, and subsequently complemented with WT *qseC* recovering WT phenotypes. We think complementation of these mutants is sufficient to prove that phenotypes are due to the described mutations and not off-target effects.

Since phosphorylation of QseB occurs in an aspartic acid we could not introduce a phosphomimetic variant in the bacteria. However, we thought it interesting to include a non-phosphorylatable variety of QseB in which the aspartic acid is exchanged by a non-phosphorylatable alanine. Changing this amino-acid in WT *E. coli* had no effect in motility, indicating that non-phosphorylated QseB is able to lead to the background suppression seen in WT *E. coli*. Introduction of this non-phosphorylatable QseB variant in a Δ*qseC* background also recovers WT phenotypes, indicating that phosphorylated QseB is the responsible for the reported phenotypes (Lines 160 to 174, Figure 4).

Comment 2. In EHEC and UPEC, QseB varies a single amino acid and swapping the two does not alter QseB activity. It would be good to provide QseB and QseC sequence alignment between UPEC, EHEC and aEPEC.

We have provided an alignment of the regulators QseB and PmrA in the supplementary data. EPEC and UPEC regulators have almost 100% identity. EHEC QseB varies 1 amino-acid from EPEC and UPEC, while PmrA varies in 3 amino-acids (Lines 361 to 365, Suplementary Figure 2).

Comment 3. One thought is that phylogeny may dictate QseBC regulation. Is the particular aEPEC strain used E, A or B2 clade?

We checked the phylo-group from aEPEC O125ac:H6 and belongs to the phylo-group B2, in a similar manner to most UPEC, while EHEC belongs to phylo-group E. The possible phylotype-dependency of the reported differences in regulation has been included in the discussion (Lines 361 to 365).

Comment 4. Provide the statistical test used for each analysis and each figure. Stating that GraphPad9 was used is inadequate.

Further statistical explanation has been provided in the manuscript (Lines 531 to 538).

<u>Reviewer 3.</u>

Comment 1. Complementation of the mutants and quick motility assay, or alternatively whole genome sequencing is needed to ensure no accumulation of secondary mutations during the genetic manipulation and that the phenotypes can be indeed attributed to the genes mutated.

To ensure that accumulation of secondary off-target mutations are not responsible for the seen phenotypes we have complemented the relevant mutants. First, we complemented Δ*qseB* with *qseB* WT gene. This strain recovered the WT phenotypes. Then, we complemented Δ*pmrB*Δ*qseC* first with WT *pmrB*, showing phenotypes similar to Δ*qseC* mutant, and subsequently complemented with WT *qseC* recovering WT phenotypes. We think complementation of these mutants is sufficient to prove that phenotypes are due to the described mutations and not off-target effects (Lines 160 to 174, Figure 4).

Comment 2. Growth curves on Figure 3 need to have the rate of duplication calculated and statistics added to claim differences.

Main differences in growth can be found during the late exponential phase. To account for these differences, the growth curves were fitted to a logistic growth function and the growth rate calculated. These constants were used to find significant differences with respect of the WT and the constants and p-values are shown in the manuscript (Lines 141 to 146, Supplementary Figure S4) .

Comment 3. In the epinephrine experiments (Figure 6 and Lines 200-216) several issues need to be addressed. They use 500 µM of epinephrine, which is overkill. Moreover, were the plates protected from light at all times? Epinephrine oxidized and is highly unstable, the stocks have to be freshly made, used and tossed. All experiments need to be light protected. This information

needs to be provided. Moreover, dopamine is NOT an inducer of QseC, if t acts it would be through other pathway.

Different concentrations of epinephrine, dopamine and AI-3, (500 nM, 5 µM, 50 µM and 500 µM) were used in the experiments. The concentration had no effect on the results so only the highest concentration was shown manuscript. To address the comments of the reviewer we added results to the in results and material and methods (Line 191 and Line 445). Experiments with epinephrine were performed protected from exposure to light. The experiments with epinephrine were repeated and confirm the results presented. Finally, we acknowledge that dopamine has not been linked to QseBC regulation and we have remove dopamine treatment results from the manuscript .

Comment 4. Experiments regarding LEE expression should not be conducted on LB, where the island is poorly expressed and regulated *in vitro.* The standard in the field for EPEC and EHEC is to express LEE in DMEM low glucose. In fact, KdpE does not regulate LEE in high glucose. Repeat LEE experiments in DMEM low glucose, and add other genes beside ler (eae, espA, etc..) to qRT-PCR and do Western of EspB and/or EspA on secrete proteins. Several investigators in the field can provide this antisera. Besides, in Figure 7A provide the actual luciferase data and add statistics.

The LEE experiments were repeated in low glucose DMEM. In this media there were no expression differences between WT and the mutants, indicating when activation occurs QseBC does not play a significant role in regulation of LEE, at least in aEPEC. This result was corroborated by qPCR for *ler* and *espA* gene expression in DMEM low glucose with WT, Δ*qseB* and Δ*qseC* strains (Line 223 to 229, Figure 7). Binding sites for QseB and PmrA were found using MEME in the *ler* promoter, suggesting that regulation may be dependent on growth medium. As we did not found a difference in regulation in DMEM low glucose we did not quantify expression of EspB or EspA by Western Blot. Figure 7A is a simplified representation of gene upregulation or downregulation. Supplementary figure 1 shows the relative luciferase expression with respect to the WT and raw luminescence data has been included in Supplementary Table S5.

<u>Academic Editor</u>

Comment 1. Genetic and *in vitro* phosphorylation are required for supporting the link between QseB and PmrB.

As referred in comment 1 from reviewer 1 we constructed non-phosphorylatable PmrB and QseB in a Δ*qseC* background. Introduction of PmrBH152A and QseBD51A into a Δ*qseC* background leads to a recovery of WT motility phenotypes indicating that in absence of QseC PmrB is the responsible of QseB phosphorylation, and that is phosphorylated QseB the responsible of the aberrant phenotypes (Lines 160 to 174, Figure 4). In addition *in vitro* phosphotransfer assays were shown before in literature in the

similar Uropathogenic *E. coli* by Guckes *et al*[1]. Given the new genetic data we provided we think the proposed *in vitro* phosphotransfer assay with PmrB and QseB is not critical.

[1]. Guckes KR, Kostakioti M, Breland EJ, Gu AP, Shaffer CL, Martinez CR, et al. Strong cross-system interactions drive the activation of the QseB response regulator in the absence of its cognate sensor. Proceedings of the National Academy of Sciences. 2013 Oct 8;110(41):16592–7.

Comment 2. DNA-protein experiments are necessary to complete the regulation model that the authors propose.

In order to show direct regulation of QseB-P of described genes we chose to prove binding of QseB-P to the conserved motif in the *arn* promoter. We found that phosphorylated QseB binds to the predicted QseB binding site in the *arn* promoter whereas the non-phosphorylated QseB binds with lower affinity. These assays corroborate that QseB directly regulates *arn* operon (Lines 265 to 269, Figure 8).

Comment 3. Experiments regarding LEE expression in the *in vitro* conditions were not performed as experimentally required.

Experiments regarding LEE expression were repeated using suggested media DMEM low glucose (see comment 4 reviewer 3) (Line 223 to 229, Figure 7).

Comment 4. Can you justify why is it important to study the effect of adrenergic signals for the pathophysiology of EPEC infection?

Adrenergic signals such as epinephrine and norepinephrine are found in the GI tract. In EHEC it has been proposed that upon reaching the human GI tracts the bacteria senses the autoinducer-3 (AI-3) produced by the intestinal flora and epinephrine and norepinephrine produced by the host through the QseBC TCS to activate motility and virulence[1] (Line 77). It was of interest then to study the sensing of these signals by QseBC and PmrAB in EPEC.

[1] Hughes DT, Clarke MB, Yamamoto K, Rasko DA, Sperandio V. The QseC adrenergic signaling cascade in enterohemorrhagic *E. coli* (EHEC). PLoS Pathog. 2009 Aug 21;5(8):e1000553.

Comment 5. Studying colistin resistance is interesting in the context described in this paper. However, you should justify such a goal in a paper deciphering TCS in EPEC. For instance, should we understand EPEC is more like to become resistant to colistin?

The TCS PmrAB has been widely linked to colistin and polymyxin resistance in various bacterial species. Therefore, we thought it was interesting to understand if cross-regulation with QseBC had any impact in polymyxin resistance regulation. Besides, it has been shown that in *Klebsiella* that point mutations in PmrAB lead to resistance to colistin. Investigation if these mutations lower PmrA affinity to colistin resistance genes allowing QseB to upregulate would be interesting (Lines 356 to 371). This

could also mean that point mutations in PmrA in EPEC may lead to colistin resistance by lowering affinity to the colistin resistance promoters .
