# Supplementary material for: Cross-talk between QseBC and PmrAB two-component systems is crucial for regulation of motility and colistin resistance in Enteropathogenic Escherichia coli
Source: PLoS Pathog. 2023 Dec 7;19(12):e1011345. doi: 10.1371/journal.ppat.1011345 (PMC10729948; doi:10.1371/journal.ppat.1011345)
Supplement: Attachment — Submitted filename: Rebuttal-Minor revisions.docx [file ppat.1011345.s009.docx]

Dear Editor and Reviewers,

We thank the reviewers for their constructive comments and suggestions which has helped to improve the quality of the manuscript. Below the reviewers comments are addressed point by point for your consideration.

**Reviewer 1**

**Comment 1. Which is the relevance of the QseB-binding site found on *arn*? It is known the transcription site for *arn*? How could be the *arn* activation by QseB-P?**

The *arn* transcription site has not been determined for *arn* operon. However, using the prediction tool BPROM we predicted the -35 and -10 sites, both adjacent to the PmrA binding site, and a rpoD17 binding site was predicted to be inside the PmrA-binding site. These predicted sequences are now shown in Figure 8. This suggests that PmrA prevents RNA polymerase from initiating transcription of the *arn* operon. The *arn* operon is constitutively expressed only when PmrA is absent and QseB is activated, indicating that phosphorylated QseB is only able to activate *arn* expression when PmrA is not bound. Due to the upstream binding site of QseB, it probably activates expression by recruiting RNA polymerase^1^. This is now discussed in lines 392 to 397 in the general discussion.

[1] Browing D. F. and Busby, S. J. W. Local and global regulation of transcription initiation in bacteria. *Nat Rev Microbiol* (2016)

**Comment 2. The authors analyse the expression of *ler* gene*,* which is the first gene of the LEE1 operon, one of the five operons that constitute the LEE island. The authors use these concepts interchangeably. Lines 242, 245, 253, 264, 259.**

Indeed, we chose *ler* since it is considered the master regulator of the LEE island. I changed all instances where *lee* is used instead of *ler,* and included an explanation on our choice for *ler* expression Lines 86, 247, 249, 252, 272, 374, 378.

**Reviewer 3**

**Comment 1. The authors failed to address the actual protein-protein interaction experiments. While elegance of the site directed mutagenesis approach adopted in the study is commendable, the authors cannot reject the hypothesis of an indirect link between PmrAB and QseBC without showing phosphorylation of QseB by PmrB in their aEPEC strain. In consequence, either these experiments should be performed or the phrasing of the manuscript should be modified to reflect lack of direct observation of protein phosphorylation. For instance, L170 “PmrB is directly responsible for phosphorylation of QseB” or L306-308 “In the WT, QseB can be phosphorylated by the non-cognate PmrB HK, but it is immediately de-phosphorylated or sequestered by QseC” are both very strong assertions when the experiment hasn’t been done by the authors in that aEPEC background.**

We have changed text to acknowledge the uncertainty of our conclusions due to the lack of direct protein-protein interaction experiments Lines 29, 199, 350, 354 to 360.

**Comment 2. L201-202 “This data suggests that PmrAB TCS has an independent role in adapting to metal ion stress that does not involve QseBC TCS”. Since the authors are studying the effect of excess iron it might be interesting to note that a Fur-box has been identified *in silico* upstream of *flhCD* in the K12 strain. If this is also the case in the aEPEC strain studied, the lack of a functional PmrAB system leading to a increase in intracellular iron (according to reference 27) could also explain the decrease of motility observed.**

It was shown in Uropathogenic *Escherichia coli* motility is increased in the presence of an iron chelator and in a *fur* mutant^2^. This suggest that excess intracellular iron would lead to repressed motility as we observed for the PmrAB two-component systems mutant when iron is present. It is indeed a possibility that is the excess of intracellular iron when the bacteria cannot respond to iron in the environment the responsible of the repression. We also see a Fur-box in the *flhCD* promoter of aEPEC, however more experiments would be needed to test this theory and that the regulation of motility in this case is through *flhCD* and no other motility genes. This point has been added in the discussion (Lines 401 to 412)

[2] Kurabayashi, K., Agata, T., Asano, H., Haruyoshi, T. and Hirakawa, H. Fur represses adhesion to, invasion of, and intracellular bacterial community formation within bladder epithelial cells and motility in Uropathogenic *Escherichia coli.* (2016) *Infect Immun*

**Comment 3. “Minimal inhibitory concentrations against aEPEC were performed as described by the European Committee on Antimicrobial Susceptibility Testing”. Is there a reason the authors chose to determine MIC in LB media instead of the recommended Mueller Hinton broth?**

It is true that MHB is the media that is used for MIC experiments, LB media was used to maintain the same medium across experiments. Experiments have been repeated with MHB media, no differences have been observed in the MIC. Line 567

**Comment 4. Figure 4b and 5, isn’t the difference between WT and the Δ*qseC* mutant significant on both graphs?**

In Figure 4b and 5 I added the relative motility of mutants Δ*qseB* and Δ*qseC* that is depicted for the first time in Figure 4a as visual help to compare double mutant relative motility to these single mutants. I added now the significance to them to avoid confusion.

**Comment 5. Typos.**

All typos were corrected. (L42, L444, Figure 7B)
